# Coupled Gold Nanoparticles with Aptamers Colorimetry for Detection of Amoxicillin in Human Breast Milk Based on Image Preprocessing and BP-ANN

**DOI:** 10.3390/foods11244101

**Published:** 2022-12-19

**Authors:** Ziqian Ye, Jinglong Du, Keyu Li, Zhilun Zhang, Peng Xiao, Taocui Yan, Baoru Han, Guowei Zuo

**Affiliations:** 1 Key Laboratory of Diagnostic Medicine Designated by the Chinese Ministry of Education, Department of Laboratory Medicine, Chongqing Medical University, Chongqing 400016, China; 2 Medical Data Science Academy, College of Medical Informatics, Chongqing Medical University, Chongqing 400016, China

**Keywords:** breast milk, amoxicillin, colorimetric methods, image preprocessing, back propagation-artificial neural network

## Abstract

Antibiotic residues in breast milk can have an impact on the intestinal flora and health of babies. Amoxicillin, as one of the most used antibiotics, affects the abundance of some intestinal bacteria. In this study, we developed a convenient and rapid process that used a combination of colorimetric methods and artificial intelligence image preprocessing, and back propagation-artificial neural network (BP-ANN) analysis to detect amoxicillin in breast milk. The colorimetric method derived from the reaction of gold nanoparticles (AuNPs) was coupled to aptamers (ssDNA) with different concentrations of amoxicillin to produce different color results. The color image was captured by a portable image acquisition device, and image preprocessing was implemented in three steps: segmentation, filtering, and cropping. We decided on a range of detection from 0 µM to 3.9 µM based on the physiological concentration of amoxicillin in breast milk and the detection effect. The segmentation and filtering steps were conducted by Hough circle detection and Gaussian filtering, respectively. The segmented results were analyzed by linear regression and BP-ANN, and good linear correlations between the colorimetric image value and concentration of target amoxicillin were obtained. The R2 and MSE of the training set were 0.9551 and 0.0696, respectively, and those of the test set were 0.9276 and 0.1142, respectively. In prepared breast milk sample detection, the recoveries were 111.00%, 98.00%, and 100.20%, and RSDs were 6.42%, 4.27%, and 1.11%. The result suggests that the colorimetric process combined with artificial intelligence image preprocessing and BP-ANN provides an accurate, rapid, and convenient way to achieve the detection of amoxicillin in breast milk.

## 1. Introduction

Human breast milk is considered the ‘perfect food’ for infants due to its natural nutrients and bioactive proteins [1,2]. In addition to nutritional functions, breast milk is an early source of bacteria introduced to the infant’s gut within a few hours of birth [3]. The breast milk microbiota could be shaped by the maternal diet [4]. Studies have shown that an altered intestinal microbiota increases the risk of allergies, asthma, and eczema [5,6]. Nevertheless, it is very common for pregnant women to use antibiotics for unavoidable reasons, such as mastitis. In a world where everyone is exposed to the overuse of antibiotics, breast milk cannot remain unaffected. Some studies have shown that intrapartum antibiotic prophylaxis (IAP) plays an essential role in the intestinal microbiota of infants [7,8]. Amoxicillin is one of the most common and widely used antibiotics today and affects the abundance of *E. coli* and anaerobic bacteria [9]. However, few studies have turned their attention to the detection of amoxicillin in human breast milk.

Regarding the detection of medical drugs in breast milk, there are fluorescence methods [10], enzyme-linked immunosorbent assays (ELISA) [11], ultra-performance liquid chromatography coupled to tandem mass spectrometry (UPLC/MS-MS) [12], high-performance liquid chromatography (HPLC) [13], etc. Although these methods have the characteristics of stability, high sensitivity, and good specificity, they are expensive and time-consuming, making wide use difficult. Therefore, there is a significant need for quick and easy biosensors to detect medical drugs in breast milk.

Nanoparticles, known for their small size and special characteristics, including colorimetric, fluorescent, and electrochemical properties, have been widely used in many fields [14]. Gold nanoparticles (AuNPs) can be a remarkable scaffold owing to not only their special properties but also their easy synthesis and excellent biocompatibility [15]. Aptamers are short single-stranded DNA or RNA oligonucleotides that are selected by SELEX (Systematic Evolution of Ligands by Exponential Enrichment) [16]. Compared to antibodies, aptamers are less expensive, easier to prepare, and more stable. Biosensors using gold nanoparticles combined with aptamers are applied in various methods, especially optical methods [17]. Colorimetry is a method in which results can be observed by the naked eye and the change can be captured by electronic devices without costly equipment. Due to their stability, high sensitivity and selectivity, cost-effectiveness, and ease of use, colorimetric methods are one of the most widely used detection method applications [18]. At the same time, colorimetric methods can also be used in conjunction with artificial intelligence algorithms, such as the smartphone-MATLAB algorithm [19], to improve the convenience and speed of detection.

There are many colorimetric assays for medical drugs that are performed by analyzing absorbance values, which not only require specific instruments but are also complex to operate. It is more convenient to directly analyze the image instead of the absorbance values. In recent years, the development of advanced photoelectric vision sensors has made it possible to recognize advanced visual information images and preprocess data. Compared with traditional computing methods, neuromorphic vision computing can significantly improve the data processing speed and energy efficiency [20,21,22]. Some image sensor arrays can simultaneously capture and recognize optical images, processing the information quickly without converting it to digital format [23]. When these sensors are performing computational tasks, there are denoising, edge enhancement, spectral filtering, and visual information recognition processes [24], which are normally performed with the help of some artificial intelligence algorithms, such as neural network algorithms. This kind of bioinspired neuromorphic vision sensor has become a major research topic in recent years, and its advantages of being smarter and faster have contributed to its wider application in various fields.

BP-ANN (back propagation-artificial neural network) is an important part of artificial intelligence algorithms, which refers to a nonlinear modeling method for data analysis to transduce imputed information [25]. BP-ANN consists of three different layers, including the input layer, hidden layer, and output layer, which can be successfully employed to imitate the enigmatic and entropic associations of the input and output signals [26]. The normal procedures of BP-ANN modeling are as follows: signals are presented to the input layer, and then the resulting output signals are transferred to the hidden layer; ultimately, the output signals are generated by the output layer [27]. BP-ANN can reduce errors based on incomplete and incorrect information to achieve a great prediction level. The results are made more accurate as the training process is continuously enhanced. Due to its excellent prediction ability, the BP-ANN has been used in a wide range of areas [28].

Here, we establish a new convenient, rapid, and accurate method for the detection of antibiotics in breast milk. Our research uses colorimetric reactions generated by the binding of gold nanoparticles to an aptamer combined with image preprocessing and BP-ANN to generate a data model between amoxicillin and color change.

## 2. Materials and Methods

### 2.1. Reagent

Aptamers (5′-TTAGTTGGGGTTCAGTTGG-3′) were synthesized and HPLC-purified by Shanghai Sangon Biological Engineering Technology and Services Co., Ltd. (Shanghai, China). Chloroauric acid (HAuCl_4_·4H_2_O), trisodium citrate, and amoxicillin (AMO) were purchased from Shanghai Sangon Biological Engineering Technology and Services Co., Ltd. Ampicillin (AMP), cefixime (CFM), cefotaxime (CEF), chloramphenicol (CHL), tetracycline (TET) and ionic salts were purchased from Shanghai Macklin Biochemical Co., Ltd. (Shanghai, China). Trichloroacetic acid (10% *w*/*v*) was purchased from Shanghai Macklin Biochemical Co., Ltd. All chemicals used were analytical reagent grade, and the solutions were prepared with ultrapure water (18.2 MΩ cm^−1^, 25 °C), which was purified with Milli-Q Integral (Millipore, Eschborn, Germany).

### 2.2. Measurements and Apparatus

Field-emission transmission electron microscopy (TEM) images of the prepared composites were obtained using a Hitachi HT-7500 TEM (Hitachi, Chiyoda, Japan). The results were scanned by a scanner (V200 PHOTO, EPSON) The experiment absorbance was achieved by an EON Microplate Spectrophotometer (BioTek Instruments Inc., Winooski, VT, USA) and processed by Gen 5 software (version 3.08.01). The UV–vis absorption spectra were obtained by Nanodrop One (Thermo Fisher Scientific, Waltham, MA, USA).

### 2.3. Synthesis of AuNPs

All the glassware required for the synthesis process was immersed in freshly prepared aqua regia (HNO_3_:HCl = 1:3) for at least 1 h, completely flushed in ultrapure water, and dried. AuNPs were prepared according to Turkevich’s method [29], and trisodium citrate (38.8 mM, 5 mL) was quickly poured into a boiling HAuCl_4_ solution (1 mM, 50 mL). The color changed from light yellow to deep purple–red, and the solution was stirred continuously throughout the synthesis process until it was cooled to room temperature. The solution was stored at 4 °C in the dark for further use. According to Lambert Beer’s law, the absorbance of 13 nm AuNPs at 520 nm wavelength is 2.7 × 10^8^ M^−1^ cm^−1^ [30].

### 2.4. Colorimetric Detection of Amoxicillin

The detection was carried out in 10 mM PBS solution and based on the following steps. In brief, 4 mL AuNPs (13 nM) was mixed with 2 mL ssDNA solution (1 µM) at a ratio of 2:1 and incubated for 30 min based on van der Waals forces. Amoxicillin solutions were prepared with PBS at concentrations of 0, 0.05, 0.1, 0.2, 0.3, 0.5, 0.7, 1.1, 1.5, 2.3, 3.1, and 3.9 µM. After 10 min of incubation of each concentration of amoxicillin solution (80 µL) with AuNPs-ssDNA (100 µL) solution, 30 µL of NaCl (1 mol/L) solution was added at room temperature. The images and UV–vis spectra of the whole solution were captured after 2 min.

### 2.5. Image Preprocessing and Data Analysis

To develop a low-cost and fast technology, a colorimetric sensor based on machine learning was used for quantitative analysis of amoxicillin in human breast milk. The key was to train the machine learning model by using the collected colorimetric images.

In this study, we preprocessed the acquired colorimetric images. Image preprocessing included three steps: segmentation, filtering, and cropping. Segmentation was performed using Hough circle detection. Hough circle detection was used to determine the target circle according to the weight of the curve intersection points in Hough space by traversing every potential circle of nonzero pixels. Because Hough circle detection is sensitive to noise, median filtering should be performed for the contrast image in advance to reduce the impact of noise on the judgment of circles. Gaussian filtering on the segmented colorimetric image effectively suppressed noise and smoothed the image. Finally, the Gaussian-filtered colorimetric images were cropped into an image of 50 × 50 size.

Linear regression and BP neural networks were jointly used for this research. The BP neural network is a multilayer feedforward neural network trained according to error backpropagation, which has a strong nonlinear mapping ability and can fit any curve. The performance of the machine learning model is evaluated by the mean square error (MSE) and correlation coefficient (R^2^) in the training set and test set. All image processing and data analysis were completed with Python 3.6 for Windows 10.

### 2.6. Preparation of Real Samples

Breast milk samples were collected from 2 breastfeeding mothers at the Women and Children’s Hospital of Chongqing Medical University after obtaining informed consent. The samples were prepared following a previous study process [31]. The process was as follows: breast milk (1 mL) was placed in a centrifuge tube, and 0.25 mL trichloroacetic acid (10% *w/v*) was added to precipitate proteins. The supernatant was filtered through a 0.22 µm ultrafiltration membrane (PVDF). The filtrate was transferred into a new centrifuge tube, and 1 mL PBS (10 mM) was added for dilution. After vortex mixing and sonication for 10 min, the sample was centrifuged at 12,000 rpm for 10 min. The supernatant was transferred into a 15 mL centrifuge tube and diluted 10 times (a 20-fold dilution).

Three different concentrations (10 µL) of amoxicillin were spiked into the prepared breast milk samples so that the final AMO concentration was 0.3, 1.0, and 3.0 µM. The detection process was conducted as described in method 2.4. All experiments were performed in triplicate.

## 3. Results

### 3.1. Design of the Experimental System

**Scheme 1:**
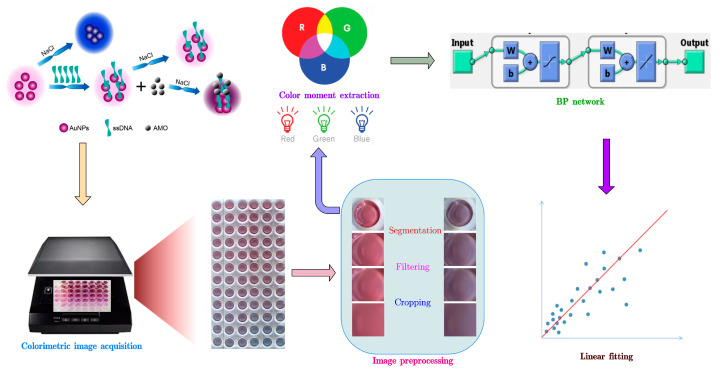
Schematic illustration of the detection of amoxicillin in breast milk. When different concentrations of amoxicillin were bound to AuNPs-ssDNA, different color reactions were produced. The results were captured by portable image acquisition equipment and processed by artificial intelligence algorithms.

**Figure 1:**
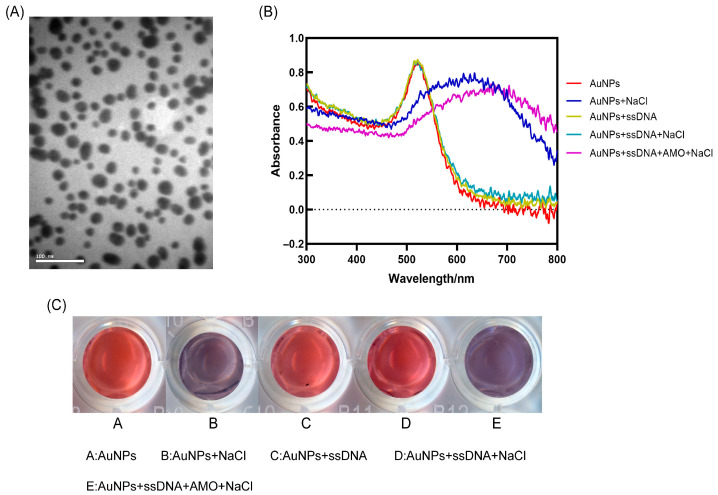
(**A**) Transmission electron microscopy results of AuNPs. (**B**) Spectral scan results when different components were present in the solution (AuNPs; AuNPs+NaCl; AuNPs+ssDNA; AuNPs+ssDNA+NaCl; AuNPs+ssDNA+AMO+NaCl). When AuNPs were dispersed, the absorption peak was around 520 nm, and the absorption peak was around 620 nm when aggregated. (**C**) Color changes when different components were present in the solution (AuNPs; AuNPs+NaCl; AuNPs+ssDNA; AuNPs+ssDNA+NaCl; AuNPs+ssDNA+AMO+NaCl).

The whole reaction process of this research is demonstrated in Scheme 1. The AuNPs we synthesized were well dispersed in the transmission electron microscopy results (Figure 1A). The dispersed AuNPs had the highest absorption peak at 520 nm, and when there was NaCl in the solution, the AuNPs aggregated and then had the highest absorption peak at 620 nm. The ratio of the absorption peak at 620 nm to that at 520 nm can reflect the aggregation of AuNPs; the larger the value is, the higher the degree of aggregation, and the smaller the value is, the lower the degree of aggregation. When ssDNA was coupled to AuNPs, it effectively prevented the aggregation of AuNPs due to the addition of NaCl. After AMO was added, ssDNA bound to AMO, the protective effect of ssDNA on AuNPs was diminished, and AuNPs aggregated again (Figure 1B). As AuNPs changed from dispersion to aggregation, the color of the solution changed from purple-red to blue-purple (Figure 1C).

When different concentrations of amoxicillin were bound to AuNPs-ssDNA, different color reactions were produced. The results were captured by portable image acquisition equipment and processed by artificial intelligence algorithms.

### 3.2. Optimization of the Conditions and Selectivity of the System

#### 3.2.1. Optimized Reaction Reagent Concentration

**Figure 2:**
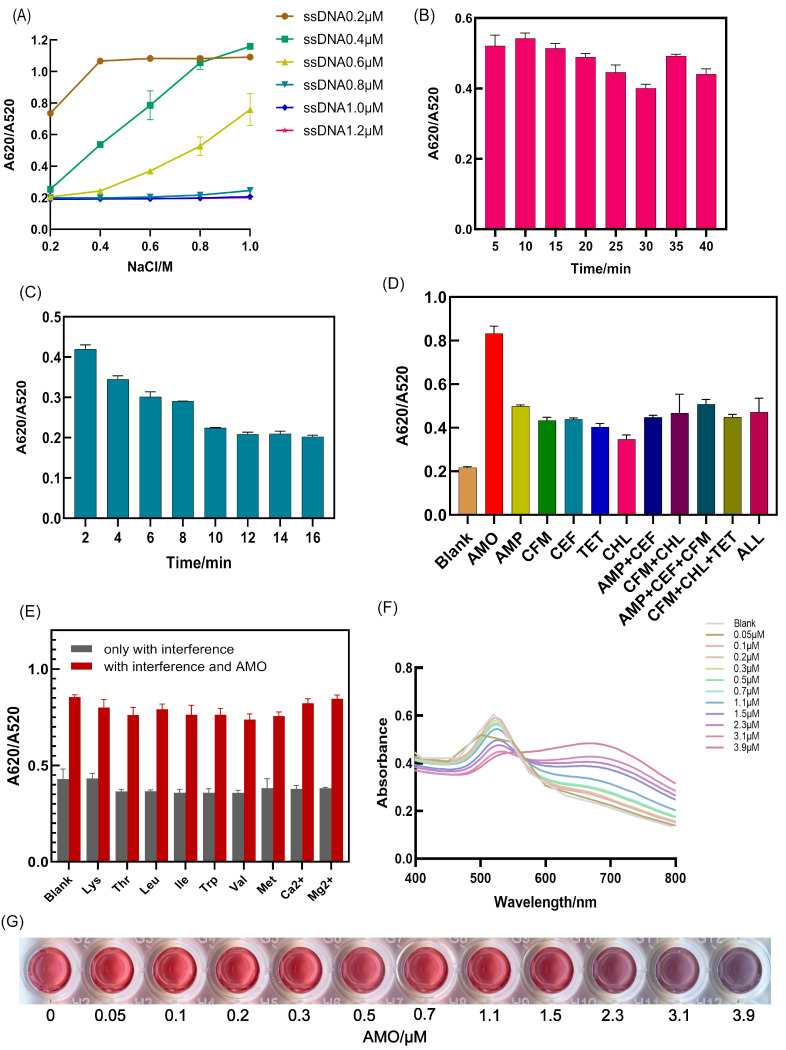
(**A**) Aggregation of different concentrations of ssDNA coupled to AuNPs in the presence of different concentrations of NaCl. (**B**) Effect of the incubation time of ssDNA with AuNPs on the protection of AuNPs by ssDNA. (**C**) Effect of reaction time between AMO and AuNPs-ssDNA. (**D**) Selectivity of the reaction system in aqueous solution. The concentrations of AMP, CFM, CEF, TET, and CHL were 6 µM, the mixture solutions were mixed at the same concentration and the concentration of AMO was 3 µM. (**E**) Interference resistance of the reaction system. Interference including amino acids and cations, the concentrations of interference used were all 50 µM and AMO was 3 µM. (**F**) Spectral scan result of the decided AMO concentrations. (**G**) Color change of the decided AMO concentrations.

The entire reaction process was carried out at room temperature. Considering the breast milk environment, PBS buffer was added to simulate the real sample environment; therefore, the pH value of the system was 7.4. Different concentrations of ssDNA coupled with AuNPs had different protective effects; the higher the concentration was, the stronger the protective effect was within a certain range. When the concentration became larger, the binding to ssDNA increased, and if the concentration of ssDNA was too low, the difference in the results was not obvious when the concentration of AMO was higher, so an ssDNA concentration of 1 µM was chosen (Figure 2A). To make the differences between the results of the various concentrations of AMO more obvious, we chose a NaCl concentration of 1 mol/L.

#### 3.2.2. Optimized Reaction Time

When ssDNA was incubated with AuNPs for a longer time, AuNPs were more protected, but if the time was too long, it would lead to the opposite effect. We verified that the optimal incubation time of ssDNA with AuNPs was 30 min (Figure 2B). In the reaction system used, too short of a reaction time between AMO and AuNPs-ssDNA would lead to the aggregation of AuNPs. We chose an optimal reaction time of 10 min (Figure 2C).

### 3.3. Selectivity of the Reaction System

In the reaction system, selectivity is an essential criterion for judging the effectiveness of the method. To verify the selectivity, some common antibiotic drugs and molecules present in breast milk, such as amino acids and cations, were selected to simulate the environment of real samples. When other antibiotic drugs, including AMP, CFM, CEF, CHL, and TET, were added, the results were different compared to AMO (Figure 2D). When the amino acid and cation interference was present in solution alone, it did not cause specific aggregation of AuNPs; however, when the interference coexisted with AMO, the aggregation of AuNPs was significantly higher (Figure 2E). All these phenomena confirmed the good selectivity of the whole reaction system.

### 3.4. Determination of the Amoxicillin Concentration Range

Considering the physiological concentration of amoxicillin in breast milk, the concentration gradient of AMO was decided from 0 µM to 3.9 µM. Different concentrations of AMO led to different aggregation abilities of AuNPs, different absorption spectra (Figure 2F), and different colors (Figure 2G).

### 3.5. Image Preprocessing and Data Analysis

**Figure 3:**
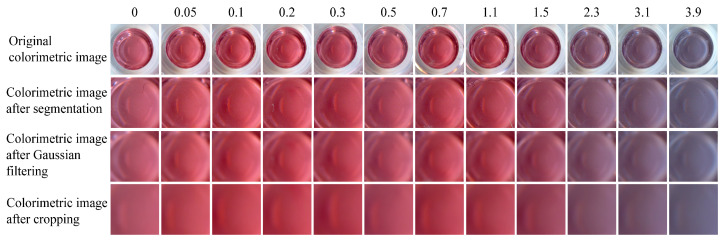
The effect of image preprocessing for the colorimetric image. Three steps were included: segmentation, Gaussian filtering, and cropping.

The effect of image preprocessing for colorimetric imaging was shown in Figure 3. In Figure 3, the original colorimetric image was finally processed into an image with a size of 50 × 50. We extracted the first-order color moment, second-order color moment, and third-order color moment from the red, green, and blue channels of the preprocessed colorimetric image. Therefore, each colorimetric image obtained 9 values to form a sample set. The first-order color moment reflected the overall lightness and darkness of the colorimetric image. The second-order color moment reflected the color distribution range of the colorimetric image. The third-order color moment reflected the symmetry of the color distribution in the colorimetric image.

**Figure 4:**
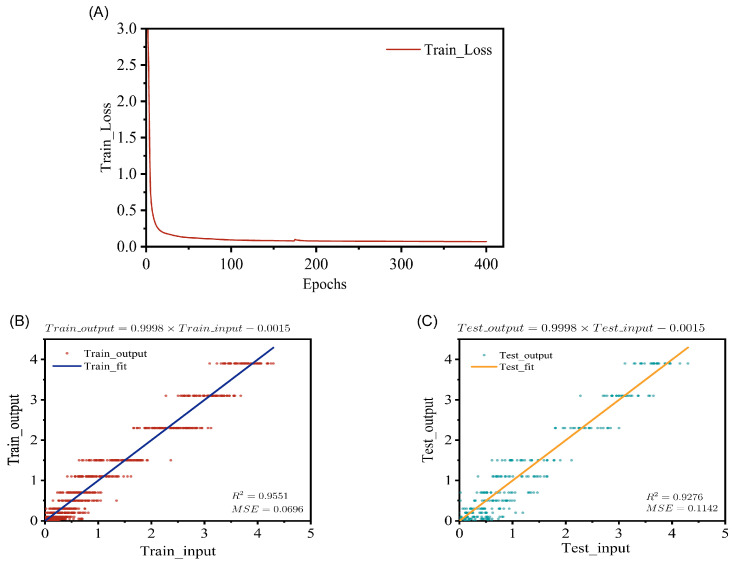
(**A**) The training curves. The maximum number of training events was 1000 epochs, and the training mean square error was 0.07. (**B**) The specific training set fitting situation. (**C**) The specific test set fitting situation.

Each group had 100 samples, and the training set and test set were divided by 3:1. The BP neural network was set as 3 layers. Since nine color moments were extracted from each colorimetric image, the number of neurons in the input layer was 9, the number of neurons in the hidden layer was 18, and the number of neurons in the output layer was 1. The learning rate factor and momentum factor were set at 0.1. The maximum number of training epochs was 1000, and the training mean square error was 0.07. The training curve is shown in Figure 4A. The training mean square error was reached after 400 epochs of training. Figure 4B,C showed that a good linear correlation between the colorimetric image value and concentration of target amoxicillin was obtained in the range of 0~3.9 µM. Figure 4B shows the specific training set fitting situation: Test_output = 0.9998 × Test_input-0.0015, MSE = 0.0696, R2 = 0.9551. Figure 4C shows the mathematical function that correlates the concentration with the response, the specific test set fitting situation: Test_output = 0.9998 × Test_input-0.0015, MSE = 0.1142, R2 = 0.9276. The limit of detection (LOD), according to described by the International Union of Pure and Applied Chemistry (IUPAC), was calculated to be 0.084 µM.

### 3.6. Detection of AMO in Real Samples

To assess the ability of our method in real samples, different concentrations of spiked amoxicillin breast milk were detected. The data are shown in Table 1. Recoveries of 111.00%, 98.00%, 100.20%, and RSD 6.42%, 4.27%, and 1.11% were achieved. According to the data, this detection method can be well applied to practical applications.

## 4. Conclusions

As described in Table 2, most detection methods have variable limits and shortcomings, in this research, a convenient, fast-monitoring, and low-cost method for the detection of amoxicillin in breast milk was successfully constructed based on gold nanoparticles (AuNPs) and aptamers coupling colorimetry and image preprocessing with BP-ANN. The consequences of antibiotics in breast milk have become an important issue in recent years, although few studies have been done, even though breast milk is a common and essential food for babies and young children. We have creatively combined the detection of antibiotics in breast milk with artificial intelligence processing for image segmentation and various treatments. Our study targeted amoxicillin in breast milk and developed a colorimetric method anchored in AuNPs and aptamers, using Hough circle detection to segment the acquired images, Gaussian filtering to suppress noise and smooth the image, and analyzing the results with BP-ANN detection methods. Compared with traditional methods, we directly capture the images and perform subsequent processing and analysis of the images. Certainly, our strategy has significant effects, which refer to less testing time and process, lower costs, and no need for complex instruments. The results can be observed by the naked eye and captured by image capture devices without any time or location restrictions. Combining neural network analysis, which has developed rapidly in recent years, with medical drug detection can help promote the application of artificial intelligence in medical research. Hence, this work has great potential for application in rapid portable and home detection in the breast milk field.

## Figures and Tables

**Table 1:** Determination of amoxicillin in breast milk samples.

Sample	Spiked (µM)	Determined (µM)	Recovery (%)	RSD (%, *n* = 3)
1	0	Not detected		
2	0.3	0.333	111.00	6.42
3	1.0	0.980	98.00	4.27
4	3.0	3.008	100.2	1.11
Mean			103.10	3.93

**Table 2:** Comparison of medical drugs detection methodology.

Methodology	Linear Range	Detection Time	Apparatus	Remarks	Ref.
FQ-EWA ^a^	200 nM–200 µM	10 min	Spectrofluorophotometer	Special apparatus	[10]
Enzyme-linked aptamer assay	0.85–32.3 µg/L	>1 h	ELISA plate reader	Complicated operation	[11]
HPLC-DAD ^b^	6.25–100 µg/mL	>1 h	HPLC system	Time-consuming and complicated operation	[13]
UPLC/MS-MS	0.12–0.32 µg/mL	>1 h	UPLC Class System	Time-consuming and complicated operation	[12]
Colorimetry	0.05–3.9 µM	10 min	Portable image acquisition device	Rapid and convenient detection	This work

^a^ Target binding facilitated fluorescence quenching; ^b^ high-performance liquid chromatography attached to a diode array detector.

## Data Availability

The data are available from the corresponding author.
